# Flexible thermoelectric generator and energy management electronics powered by body heat

**DOI:** 10.1038/s41378-023-00583-3

**Published:** 2023-08-24

**Authors:** Shuai Yang, Yumei Li, Ling Deng, Song Tian, Ye Yao, Fan Yang, Changlei Feng, Jun Dai, Ping Wang, Mingyuan Gao

**Affiliations:** 1 College of Engineering and Technology, Southwest University, 400716 Chongqing, China; 2 Chongqing Key Laboratory of Agricultural Equipment in Hilly Area, 400716 Chongqing, China; 3 Gies College of Business, University of Illinois at Urbana–Champaign, Champaign, IL 61820 USA; 4 Department of Orthopedics, Shanghai Key Laboratory for Prevention and Treatment of Bone and Joint Diseases, Shanghai Institute of Traumatology and Orthopedics, Ruijin Hospital, Shanghai Jiao Tong University School of Medicine, 200025 Shanghai, China; 5 School of Mechatronical Engineering, Beijing Institute of Technology, 100081 Beijing, China; 6 School of Civil Engineering, Southwest Jiaotong University, 610031 Chengdu, China

**Keywords:** Electrical and electronic engineering, Structural properties

## Abstract

Uninterrupted, efficient power supplies have posed a significant hurdle to the ubiquitous adoption of wearable devices, despite their potential for revolutionizing human‒machine interactions. This challenge is further compounded by the requirement of these devices to supply dependable energy for data-intensive sensing and transmission. Traditional thermoelectric solutions fail to deliver satisfactory performance under conditions of extremely low voltages. Here, we present a novel solution of a wearable thermoelectric generator integrated with an energy management system, which is capable of powering sensors and Bluetooth by harnessing body heat. Distinct from previous works, our innovation lies in its ability to consistently operate even with a minimal temperature difference (i.e., 4 K) between the human skin and the ambient environment, ensuring reliable data transmission within a time as short as 1.6 s. Furthermore, our system can recharge utilizing body heat under ultralow voltage conditions (30 mV). Our developed system provides a novel pathway for the continuous, reliable monitoring of self-contained wearable devices without depending on batteries.

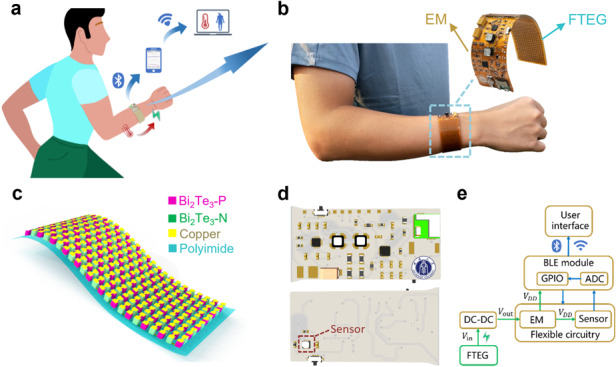

## Introduction

In recent years, the development of Internet of Things (IoT) technology has led smart wearable devices to attract worldwide attention due to their potential for flexibility, real-time information sharing, and portability^1,2^. Smart wearable devices can continuously monitor the wearer’s physiological activities and have very broad application prospects in the fields of noninvasive clinical diagnostics and real-time health monitoring^3,4^. Different from conventional bulky rigid wearable devices, flexible thin-film wearable devices can be closely attached to nonflat surfaces to achieve high-accuracy data collection, which greatly expands the application scope of wearable devices in the medical and monitoring fields^5,6^. The application of smart wearable devices has greatly promoted deeper human-machine interactions, thereby promoting the transformation of more broadly applying this sensing technology in new information-based systems^7^.

At present, standard power supply schemes for wearable devices are dominated by electrochemical batteries, especially lithium-ion batteries^8,9^. However, lithium battery electrode materials are rare and precious metals with high cost. Moreover, lithium battery electrolytes are flammable organic solvents and corrosive electrolyte salts, which present a danger of leakage or even explosion, rendering them a higher risk in fields such as implanted medical devices and vital sign monitoring. In addition, rigid Li-ion batteries are also not conducive to the flexible integration of wearable systems^10,11^. Therefore, autonomous and green power supplies have become a popular research topic in the field of wearable devices. According to its energy conversion mechanism, power supply techniques are divided into photoelectric conversion (photovoltaic cells)^12^, chemical energy conversion (fuel cell and humidity power generator)^13,14^, kinetic energy conversion such as piezoelectric nanogenerator (PENG)^15^, electromagnetic, and triboelectric nanogenerators (TENG)^16^, and electromagnetic field energy conversion (near-field wireless power transfer and far-field radio frequency energy harvesting)^17^.

These currently demonstrated green power supply methods have limitations when applied to wearable health monitoring devices. For example, photovoltaic power generation is greatly affected by ambient light conditions, and the energy density is low on cloudy and rainy days^18^. Kinetic motion energy conversion methods such as TENGs and PENGs require intensive body motion to convert mechanical energy into electrical energy, which is not suitable for elderly and disabled individuals with weak mobility^19^. Chemical energy to electrical energy conversion methods such as fuel cells require an external fuel supply, which requires regular maintenance and leads to potential safety hazards such as leakage and explosion^20,21^. The near-field wireless power supply mode has a short working distance, and the energy that can be collected by the RF far-field energy harvesting technology is very weak (e.g., at the microwatt level), which cannot power Bluetooth and other milliwatt-level devices^22,23^.

Because the human body is a stable heat source, if the temperature difference between the body heat and the environment can be used to generate electricity, such a device would not depend on external environmental conditions, relative motion, or electromagnetic radiation sources^24–27^. Numerous prior studies have thoroughly discussed the preparation of materials and device design for thermoelectric generators (TEGs)^28,29^. These studies aim to enhance the output performance of TEGs and to facilitate efficient on-chip thermal management^30^. They have led to the development of a diverse array of thermoelectric devices^31^. Such devices include thermoelectric fibers, thin films, and particle-based variants^32,33^, all of which display impressive thermoelectric properties. These advancements hold considerable promise for extensive applications in the realm of wearable electronics^34,35^.

Nevertheless, there are limitations in both current bulky thermoelectric devices and emerging flexible thermoelectric generators^36,37^. Existing demonstrations are only able to generate significant power under conditions of large temperature differences, resulting in a majority of their applications being restricted to lighting LEDs (as presented in Table S1). Moreover, at smaller temperature differences, these devices produce extremely low output voltages and power, which are insufficient to operate sensors and wireless transmission modules required in wearable scenarios^33,38^.

This paper presents a wearable health monitoring bracelet powered by body heat. The main contributions of this work are as follows: (1) Innovative energy management: we utilize an energy management (EM) circuit and supercapacitors to efficiently control and harness the inconsistent output from TEGs, significantly improving their usability and allowing operation at lower temperature differences. (2) System integration and general applicability: we integrate thermocouples and EM electronics on the same polyimide (PI) substrate, which mitigates interface effects and enhances the normalized power density of the FTEG, improving the energy conversion capability. Our system design, which starts at only 30 mV, extends compatibility across diverse thermoelectric devices and eases the deployment of self-powered wearable health monitoring systems.

The bracelet integrates a wearable thermoelectric generator, wearable energy management electronics, a Bluetooth low energy (BLE) wireless chipset, sensors, and peripheral electronics. The flexible thermoelectric generator captures the temperature potential energy across the disparate skin and ambient temperatures and converts it into electrical energy. The energy management circuit efficiently manages the harvested low-grade voltage and micro-energy so that the bracelet can work stably and power the sensor and BLE at a temperature difference as low as 4 K with a bending radius of 30 mm. The system conducts battery-free real-time monitoring with a transmission cycle as short as 1.6 s. This work indicates low voltage energy harvesting driven by body heat and justifies a self-contained wearable system for unintermittent condition monitoring by Bluetooth wireless transmission.

## Results and discussion

### FTEG + EM-powered self-contained wearable bracelet

In the field of human health monitoring, making full use of the human body’s characteristics to design and fabricate self-powered devices to provide energy for wearable devices is a current area of research interest. Capturing human body heat and converting it into electrical energy has the advantages of being environmentally friendly and sustainable. Human health monitoring also needs to be continuous, i.e., collect data 24 h per day and upload it to a cloud database for real-time dynamic monitoring of the body. At the same time, to ensure the accuracy of the collected data and long-term wearing comfort, the wearable health monitoring device should have good flexibility.

**Fig. 1:**
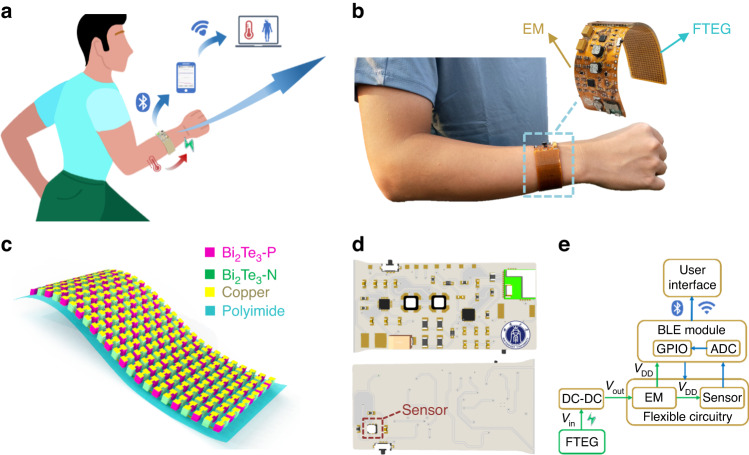
FTEG + EM-powered self-contained wearable bracelet. **a** Illustration of a wearable bracelet that integrates a flexible thermoelectric generator (FTEG) for body heat energy harvesting, energy management (EM) electronics, a thermometer for measuring body temperature, and Bluetooth-based wireless data transmission to a mobile user interface. **b** Optical images of the bracelet that can be worn on a human wrist. The inset shows images of the FTEG and EM. **c** Schematic diagram of the FTEG with P- and N-type Bi_2_Te_3_ thermoelectric chips on a polyimide substrate. **d** Schematic diagram of the flexible EM electronics, sensors, and Bluetooth modules. **e** Block diagram at the system level displaying the wireless transmission, signal processing, power management, and signal transduction of the bracelet from the FTEG to the sensors and the user interface

This paper proposes a wearable health monitoring bracelet by using body heat as the power source (Fig. 1a). The bracelet can be worn on the wrist (Fig. 1b) and features a flexible thermoelectric generator (FTEG), wearable energy management (EM) electronics, a BLE wireless chipset, sensors, and peripheral electronics (the prototype is shown in the inset of Fig. 1b). Bismuth telluride-based alloy material is the preferred thermoelectric material with good performance at room temperature. Bi_2_Te_3_ thermoelectric particles were selected as basic building blocks of the FTEG to establish the temperature difference between the skin and the environment to generate electricity. P-type and N-type Bi_2_Te_3_ particles were staggered, a polyimide (PI) film was used as a flexible substrate, and the bottom and top PI were connected with copper electrodes to form a π-type structure to realize the electrical connection of the FTEG (Fig. 1c). A total of 287 pairs of Bi_2_Te_3_-P and Bi_2_Te_3_-N thermoelectric particles were arranged on the PI film with a size of 30 mm × 80 mm (Fig. S1), and the FTEG had good flexibility and was closely attached to the skin of the human wrist for efficient thermoelectric energy harvesting. The FTEG is connected to flexible EM electronics (Fig. 1d), and the EM can convert, store and distribute the input micro-energy; that is, the EM boosts the input millivolt voltage into a standard DC voltage of 3.3 V/5 V and supplies power to the sensor and the BLE wireless transmission module under the control of an integrated chipset (IC). The sensor collects skin temperature in real time and wirelessly transmits it to the developed health monitoring mobile app terminal through a Bluetooth link to realize real-time human body temperature monitoring (Fig. 1e). The flexible circuit uses the PI film as the substrate and thus realizes the full flexible integration of the FTEG, EM, sensors, BLE, and peripheral electronics (Fig. S2). The proposed health monitoring bracelet with the EM matches the power generation performance of the generator so that the FTEG can work stably at a lower temperature difference, overcoming the limit of battery maintenance in traditional wearable devices (Supplemental Videos 1 and 2).

### Design and simulation of the FTEG

**Fig. 2:**
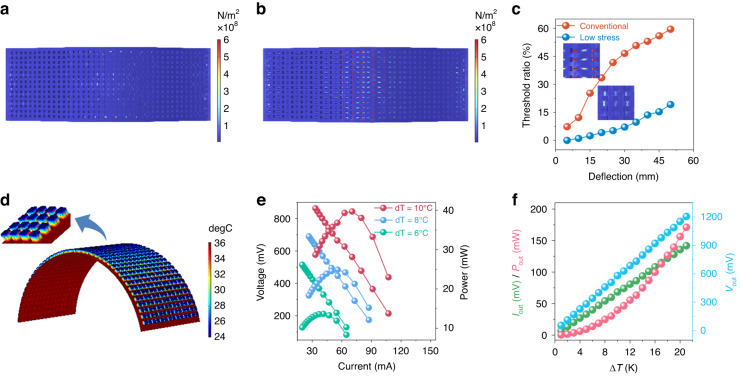
Design and simulation of the FTEG. Stress contours of the proposed low-stress electrode arrangement (**a**) and the conventional configuration (**b**) of the FTEG. **c** Comparison of the stress threshold ratio of two electrode arrangements in relation to the deflection of the FTEG. **d** Temperature distribution of the bent FTEG at $\Delta$*T* = 12 K. The inset shows the local enlargement view with red arrows indicating the direction of the heat flow. **e** Voltage and power in relation to the current for the FTEG at different temperature gradients. **f** Power, voltage, and current generated by the FTEG as a function of the temperature difference between the hot and cold sides

Current flexible thermoelectric generators do not typically focus on the stress flow in the arrangement of device electrodes. In practice, flexible devices need to be bent during use. P- and N-type thermoelectric particles and electrodes are subject to large changes in stress during the bending process. Therefore, we propose a low-stress electrode arrangement after analyzing the conventional electrode arrangement from the perspective of stress flow. The conventional electrode arrangement is shown in Fig. S3a, and our low-stress electrode arrangement is shown in Fig. S3b. The stress simulation model is shown in Fig. S3c, d. The stress distribution of the two electrode arrangements of the FTEG under different deflections was compared, the stress threshold of copper electrodes was set, and the ratio of the number of electrodes to the total number of electrodes under different deflections of the FTEG was calculated separately (Fig. 2a, b). The threshold ratio of the conventional electrode arrangement is significantly higher than that of the electrode arrangement used in this work (Fig. 2c). It is evident from the stress distribution diagram that the conventional electrode arrangement is subject to greater stress in the bent state, which is not conducive to wearable scenarios that require high flexibility. Therefore, when designing flexible wearable devices, stress analysis should be carried out according to the usage conditions so that the device can work in a low-stress state and improve the device’s durability.

To further explore the power generation performance of the FTEG, a simulation model of the FTEG is established (Fig. S4a). The temperature distribution of the FTEG at $\Delta$*T* = 12 K is shown in Fig. 2d, where the red arrow indicates the direction of heat flow. According to the external load changes (Fig. S4b), the output voltage and current curves are obtained (Fig. S4c, d). The output voltage and power of the FTEG vary with the output current under different temperature differences (Fig. 2e). The maximum output power of the FTEG could exceed 1 mW at a temperature difference of 2 K (Fig. 2f), showing a good power supply performance.

### Performance of the EM electronics at a small temperature difference

**Fig. 3:**
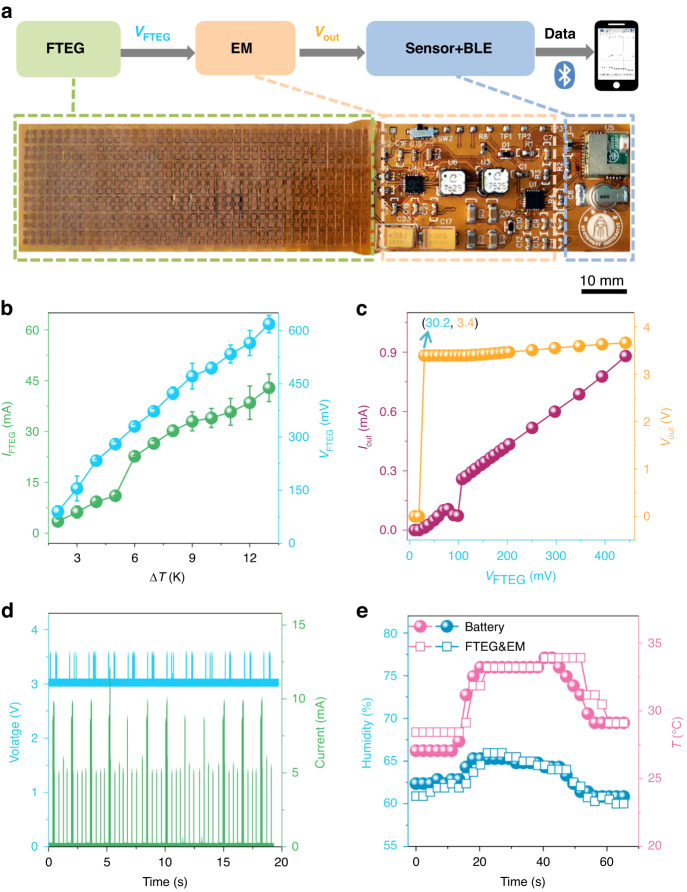
Performance of the EM electronics at a small temperature difference. **a** System architecture and photo of the self-contained wearable device powered by the FTEG plus EM. Scale bar: 10 mm. **b** Generated voltage and current of the FTEG as a function of the temperature difference between human skin and the environment. **c** Output voltage and current of the EM as a function of the generated voltage of the FTEG. When the FTEG outputs a low voltage of 30.2 mV, the EM can output a DC level of 3.4 V. **d** BLE load voltage and load current with the FTEG at a temperature difference of 2 K. The BLE transmission interval is 1.6 s. **e** Real-time data of body temperature and humidity from the wearable system powered by the FTEG plus EM at a temperature difference of 2 K. As a comparison, it shows sensor data from the system charged by a lithium battery

With complete and flexible integration of the FTEG including back-end energy management, data acquisition, and wireless transmission electronics, a flexible bracelet suitable for human health monitoring was developed (Fig. 3a). The FTEG functions based on the temperature difference between the skin and the ambient temperature, and its output current and voltage increase with the temperature gradient (Fig. 3b). In a normal state, when the temperature difference is 2 K, the FTEG delivers a stable output voltage of 89 mV and current of 3 mA, realizing a reliable power supply under this small temperature difference. The FTEG output polarity is connected to the energy management system. When the FTEG output voltage is higher than 30 mV, the EM can boost the low voltage of the FTEG into a usable DC voltage of 3.3 V/5 V (Fig. 3c), and the EM also features an automatic polarity-matching topology (Supplemental Video 3). Regardless of whether the polarity of the output voltage of the FTEG is positive or negative, the EM can work normally, which solves the problem of the polarity reversal of the output voltage caused by the reversal of the cold and hot sides of the FTEG (i.e., even if the ambient temperature is higher than the human body temperature, as long as the temperature difference reaches 2 K, the FTEG with EM can function well).

By adjusting the value of the energy storage capacitor (Figs. S5 and S6) in the EM, the system discharge rate can be adjusted to provide stable power for the data transmission module. The supercapacitor equipped in the EM can collect and store excess energy, realize an auxiliary power supply when necessary, and improve the working reliability of the system. The bracelet uses an ultralow power Bluetooth module, and we designed and developed an app to receive and monitor the collected human body temperature data in real time. When the Bluetooth module transmits data wirelessly, the time evolutions of the instantaneous voltage and current change curves are shown in Fig. 3d. The power consumption is approximately 1.5 μA in the sleep state, demonstrating that the whole system works with extremely low power consumption, while the power consumption approaches 10 mA when the BLE conducts wireless data transmission. Under a temperature difference of 2 K, the system enabled by the EM can conduct real-time monitoring with a transmission cycle as short as 1.6 s (Fig. S7 indicates the characterization and testing method for evaluating the performance of thermoelectric energy management electronics). The conventional health monitoring bracelet is powered by batteries, while the proposed bracelet relies on body heat for the power supply. To evaluate the performance of the FTEG + EM system, a comparison test is carried out, and the results are shown in Fig. 3e. The FTEG plus EM system powered by body heat can stably conduct temperature and humidity data sensing and wireless data transmission and can quickly charge and discharge to achieve continuous data sampling. The power performance is comparable to that of battery-powered devices. It not only solves the problems of troublesome maintenance and potential safety hazards of chemical batteries but also has the characteristics of being clean, green, and sustainable, contributing to the implementation of a green power supply for flexible wearable devices.

### FTEG wearability testing

**Fig. 4:**
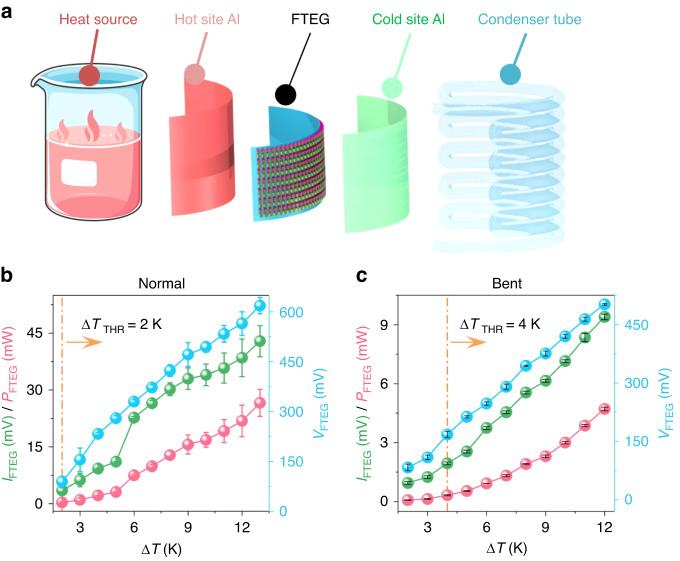
FTEG wearability testing. **a** Illustration of the wearability test of the FTEG. **b** Generated power, voltage, and current of the FTEG in a normal state as a function of the temperature difference between the hot and cold sides. The temperature gradient threshold $\Delta$*T*_THR_ for the FTEG in a normal state to enable the sensor and BLE is 2 K. **c** Generated power, voltage, and current of the FTEG in a bent state as a function of the temperature difference between the hot and cold sides. The temperature gradient threshold $\Delta$*T*_THR_ for the FTEG in a bent state to enable the sensor and BLE is 4 K

To assess the performance of the FTEG in a practical application, we constructed a flexible thermoelectric test platform (Fig. 4a). A water bath heating platform and a beaker served as heat sources, while a condenser tube facilitated cooling at the cold end. Aluminum foils were affixed to the cold and hot ends to ensure uniform temperature distribution across the FTEG. The temperature difference between the hot and cold ends was regulated by adjusting the water bath and condensate temperatures (Fig. S8).

We conducted thermoelectric performance tests on the FTEG in both a flat and bent state, while varying the temperature differences (Fig. 4b shows the data in the flat state, and Fig. 4c shows the data in the bent state). The bending radius used was 30 mm, which corresponds to the average radius of an adult male wrist^27,39^. By comparing the thermoelectric performance data between the flat and bent states, we observed that the internal resistance of the FTEG in the flat state was approximately 15 Ω when the temperature difference exceeded 5 K. However, in the curved wearing state, the internal resistance increased to approximately 60 Ω. Therefore, the output power of the FTEG in the bent state was reduced by approximately four times compared to the unbent state.

The as-fabricated FTEG includes hundreds of P- and N-type thermoelectric particles and copper electrodes, with the device made using a reflow soldering process. The contact resistance at the solder joints could be large after bending, resulting in a drop in output power. Through dynamic impedance matching, the power output capability of the FTEG in the bending state is enhanced. In the flat state, the body heat-powered bracelet can drive the entire system to work stably under a temperature difference of 2 K (i.e., an output voltage of 89 mV and current of 3 mA in Fig. 4b), and in the wearing state, the system also works stably under a temperature difference of 4 K (i.e., an output voltage of 167 mV and current of 1.9 mA in Fig. 4c), demonstrating good applicability for powering wearable devices.

Figure S9 highlights the superior power density of our device compared to other similar thermoelectric particle types. Despite many existing devices focusing on powering LEDs and requiring substantial temperature differences, our fully integrated device, which incorporates multiple thermocouples, achieves higher voltage output even with minor temperature variations. This is made possible by an efficient energy management circuit design, ensuring a reliable power supply for both the sensor and BLE (Table S1). As a result, self-powered health monitoring is demonstrated as a practical reality, representing a significant advancement in wearable thermoelectric generators. Additionally, our micro-energy management circuit only requires a minimum of 30 mV to operate, allowing compatibility with a wide range of thermoelectric devices that generate over 30 mV of output voltage (Table S2). This adaptability is crucial for the practical implementation of future advancements in cutting-edge flexible TEGs.

## Conclusion

In this study, we presented a fully flexible body heat-driven self-powered health monitoring device, which features a flexible thermoelectric generator and energy management electronics. The FTEG captures the thermal energy of the human body and converts it into electrical energy, and the EM tailored for the FTEG can achieve efficient energy management, powering of the back-end sensor and wireless transmission modules, and stable real-time human body temperature monitoring. In addition, we conducted an in-depth analysis of the FTEG electrode layout from the perspective of stress flow and incorporated a low-stress electrode layout. Compared with the traditional π-type structure, the electrode stress is significantly reduced in the wearing state, which significantly improves the device’s reliability. We demonstrated that the developed FTEG and EM can power the sensor and BLE at temperature differences of 2 and 4 K in normal and worn states, respectively. The demonstrated body-heat-driven flexible thermoelectric generator and energy management system enable reliable uninterrupted health status monitoring for IoT-enabled self-awareness scenarios, offering an opportunity for reliable and more environmentally friendly wearable devices. We note that the demand for safer thermoelectric materials in wearable device applications will drive future research toward enhancing biocompatibility and improving device structure design^40^. Additionally, it is worthwhile to explore the adaptability of the proposed energy management approach to enhance the practical application of state-of-the-art FTEG devices, such as advanced thermoelectric fibers and particles, for real-time health monitoring.

## Methods

### Design of the FTEG

We designed and customized P- and N-type thermoelectric particles with a size of 1.1 mm × 1.1 mm × 1.4 mm (Fig. S1). The PI substrate undergoes the printed circuit fabrication technique to etch patterned copper electrodes, which are subsequently gold-plated to enhance their electrical conductivity. To enhance the wettability and adhesion of the bismuth telluride thermoelectric particles, a nickel coating is applied to both the top and bottom surfaces. Based on the findings from the low-stress FTEG electrode configuration, copper electrodes measuring 1.1 mm × 1.1 mm are deposited onto the PI substrate. Solder paste is then applied to the pads on the flexible substrate. P-type and N-type thermoelectric particles are alternately positioned on the substrate pads, forming a π-type structure. Subsequently, the flexible substrate is placed in a reflow oven for welding. Furthermore, solder paste is added to the top of the folded P- and N-type particles, and the assembly is once again subjected to the reflow oven to establish electrical connections for the FTEG. Finally, the excess portion of the top PI cover is removed from the welded FTEG, resulting in a fully functional flexible FTEG (Fig. S2).

### Thermoelectric modeling and simulation

The three-dimensional model of the FTEG was established by SolidWorks 2019 and imported into COMSOL Multiphysics 6.0. Stress–strain simulation analysis was performed on the two different electrode arrangements (Fig. S3c, d). In COMSOL Multiphysics, we used the thermoelectric effect and circuit modules for steady-state simulation. The circuit node was used to connect the ammeter, the external load, the FTEG, and the voltmeter, and thus, we built the circuit simulation model (Fig. S4). By changing the resistance value of the external load, data from the ammeter and the voltmeter were collected.

### Design of thermoelectric energy management electronics

A schematic diagram of the energy management electronics is shown in Fig. S5. The schematic diagram was drawn in the circuit design software LCEDA, the connection relationship between each module was clarified, and each component was numbered reasonably. The routing was generated according to the schematic diagram, and the bill of materials (BOM) of the electronics was obtained (Fig. S6).

### Characterization and testing of thermoelectric energy management electronics

The interface design encompasses two key aspects: the thermoelectric device itself and the integration test of the TEG and the EM. To assess the overall system performance, we employed a flexible substrate with designated test points to integrate the TEG and energy management electronics, as illustrated in Fig. S7. The green solid line box indicates the positive and negative test points for measuring the FTEG output voltage and current. The DC output voltage, post energy management system, is tested by connecting DC+ and ground, while the current is measured by incorporating a digital multimeter in series with the yellow dotted line box (excluding the 0-ohm resistor during testing). Similarly, the load voltage and current of the BLE can be obtained using the same methodology. By employing the aforementioned testing approach, we can acquire real-time operational voltage and current values, enabling characterization and performance assessment of the thermoelectric energy management system.

### Data processing

Error bars, representing the standard deviation (SD), were employed. We calculated the average and standard deviation of the data from three parallel experiments using the following equation:

1 $$\bar{x}=\frac{\left({x}_{1}+{x}_{2}+...+{x}_{n}\right)}{n}$$

2 $${{\rm{SD}}}=\sqrt{\frac{{\sum }_{i=1}^{n}{\left({x}_{i}-\bar{x}\right)}^{2}}{n-1}}(n=3)$$

In the graph, each data group is represented by the average ($\bar{x}$), while the error bars represent the SD of each group. These error bars indicate the variation in the data points. Smaller error bars indicate less deviation of the data group from the mean, indicating more reliable data.

### BLE firmware and app software development

Based on the electrical characteristics of the FTEG, a low-power, small-size wireless sensor monitoring circuit was designed (i.e., Bluetooth chipset CYBLE-022001-00 and sensor SI7006). The sensor collects temperature data, and BLE transmits the collected data wirelessly to a developed app to form a micro-Internet of Things (IoT). First, we designed the wireless sensor monitoring circuit by LCEDA, connected the BLE-USB bridge and the debug board to the BLE and a computer with Win10 OS, and used the RDK Software to burn the program of the communication protocol into the BLE chip. We developed an Android app in Visio Studio using the C++ development language. The connection between the BLE and the app adopts the broadcast mode to reduce wireless transmission power consumption. Finally, we configured the app on an Android cellphone and performed the test.

### Wearability test

To test the performance of the FTEG, a flexible test platform with a controllable temperature difference was built (Fig. S8). The bending radius is 30 mm, taken from the average adult male wrist radii^27,39^. We utilized glassware equipped with a heat-insulating cover of the same radius. Inside the glassware, water at a specific temperature was added, creating a heated glass container through the use of a water bath. The hot end of the FTEG was then attached to the glass container. To ensure thermal conductivity, an aluminum foil slightly larger than the FTEG size was positioned between the hot end of the FTEG and the glass container surface. Similarly, an aluminum foil of matching size was affixed to the cold end of the FTEG, with the condenser tube connected to this foil.

The glass container, along with the attached FTEG, was placed on a heating platform within a water bath maintained at a constant temperature. To control the temperature at the cold end of the FTEG, a specific temperature of condensed water was introduced into the condensing tube. Temperature probes from the Fluke 52-II thermometer were placed on both the cold and hot aluminum foils to collect temperature data. Furthermore, the Keithley 6500 digital multimeter was connected to the FTEG to collect current and voltage data.

### Supplementary information

Supplemental Video1

Supplemental Video2

Supplemental Video3

Supplemental Material

## Data Availability

Further information and requests for resources and original data should be directed to and will be fulfilled by the lead contact, M.G. (goalmychn@gmail.com).
