# Supplementary material for: Flexible thermoelectric generator and energy management electronics powered by body heat
Source: Microsyst Nanoeng. 2023 Aug 24;9:106. doi: 10.1038/s41378-023-00583-3 (PMC10449853; doi:10.1038/s41378-023-00583-3)
Supplement: Supplementary file 4 — Supplemental Material [file 41378_2023_583_MOESM4_ESM.docx]

**Supplemental information**

**Flexible thermoelectric generator and energy management electronics powered by body heat**

Shuai Yang^1,2^, Yumei Li^1,2^, Ling Deng^1,2^, Song Tian^1,2^, Ye Yao^3^, Fan Yang^4^, Changlei Feng^5^, Jun Dai^5^, Ping Wang^6^, Mingyuan Gao^1,2*^

^1^College of Engineering and Technology, Southwest University, Chongqing 400716, China

^2^Chongqing Key Laboratory of Agricultural Equipment in Hilly Area, Chongqing400716, China

^3^Gies College of Business, University of Illinois at Urbana–Champaign, Champaign IL 61820, USA

^4^Department of Orthopedics, Shanghai Key Laboratory for Prevention and Treatment of Bone and Joint Diseases, Shanghai Institute of Traumatology and Orthopedics, Ruijin Hospital, Shanghai Jiao Tong University School of Medicine, Shanghai 200025, China

^5^School of Mechatronical Engineering, Beijing Institute of Technology, Beijing 100081, China

^6^School of Civil Engineering, Southwest Jiaotong University, Chengdu 610031, China

*Corresponding author: [goalmychn@gmail.com](mailto:goalmychn@gmail.com)

Supplemental information includes Supplemental Figures S1-S9 and Supplemental Tables S1-S2.


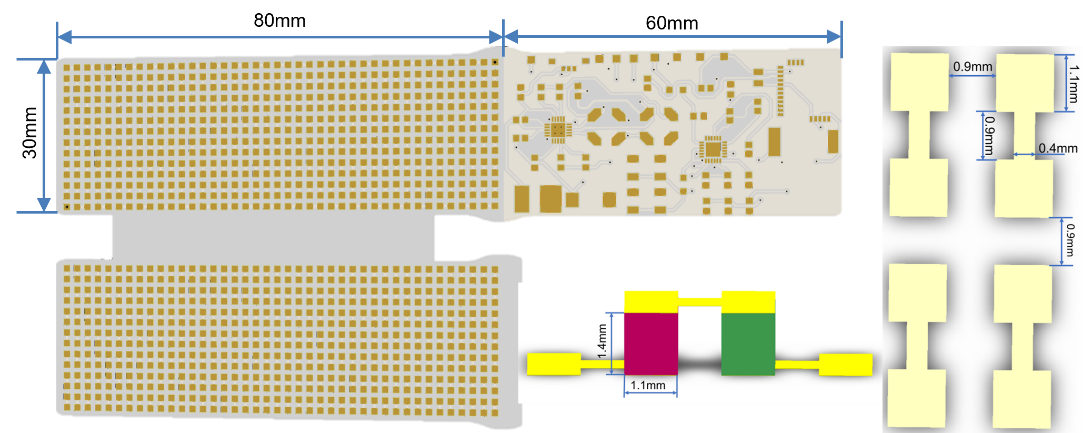


Figure S1. Illustration of the electrode layouts and dimensions of the device.


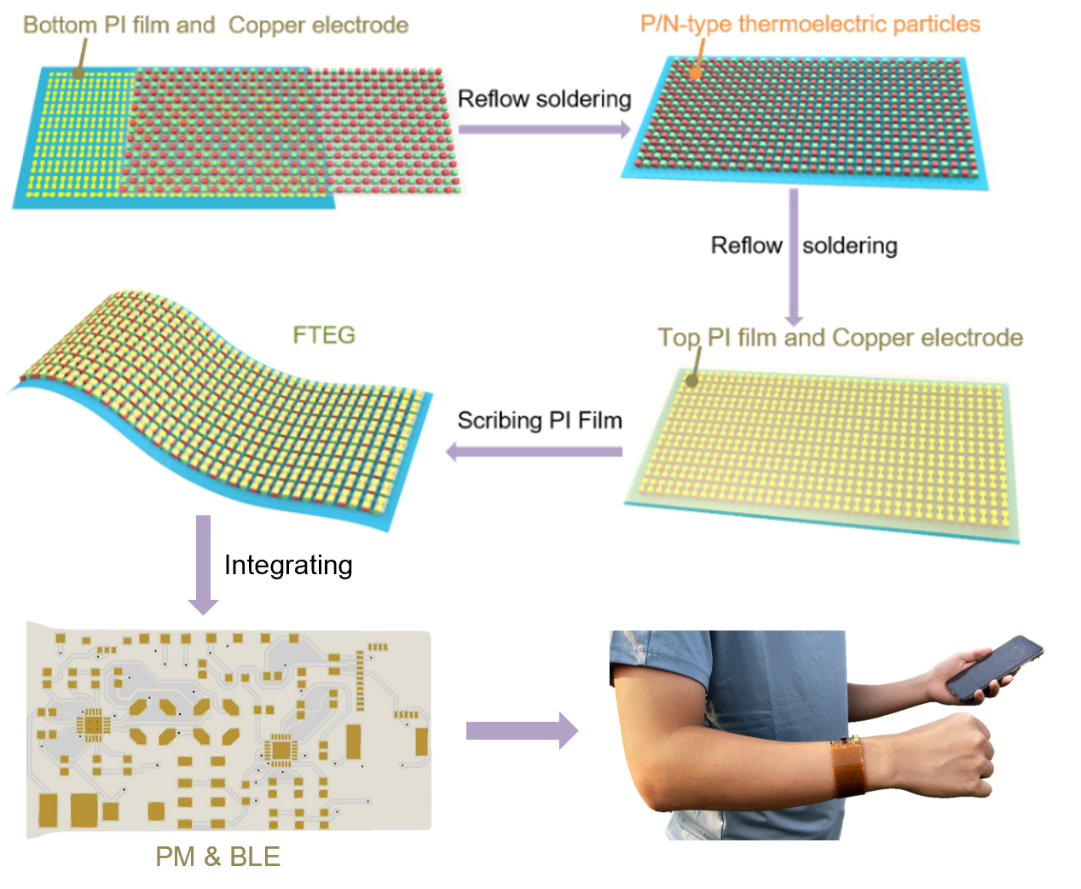


Figure S2. Fabrication process of the FTEG.


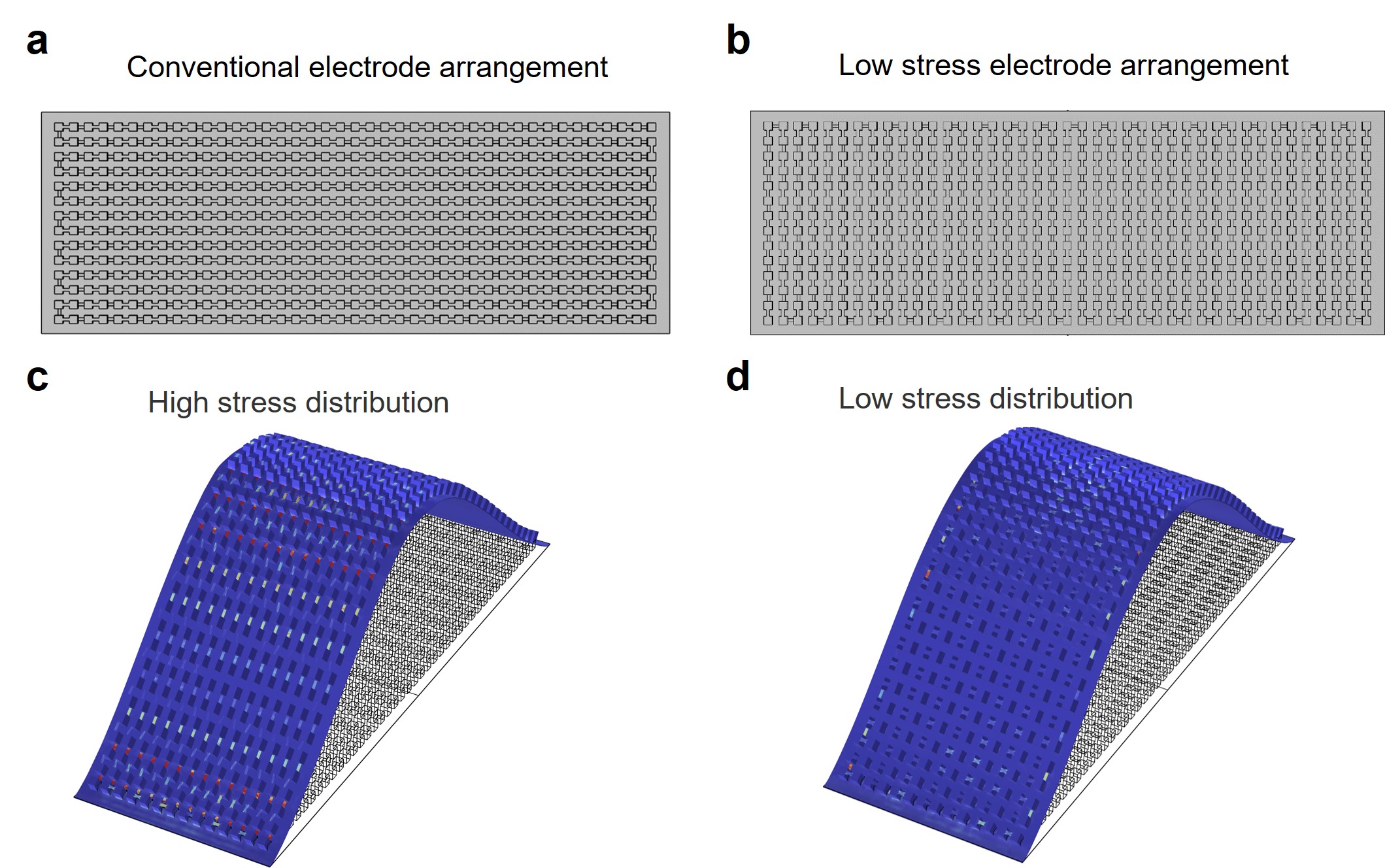


Figure S3. Stress simulation model of the FTEG. (a) Conventional electrode arrangement. (b) Low-stress electrode arrangement. Stress contour of the (c) conventional electrode and (d) low-stress electrode configurations.


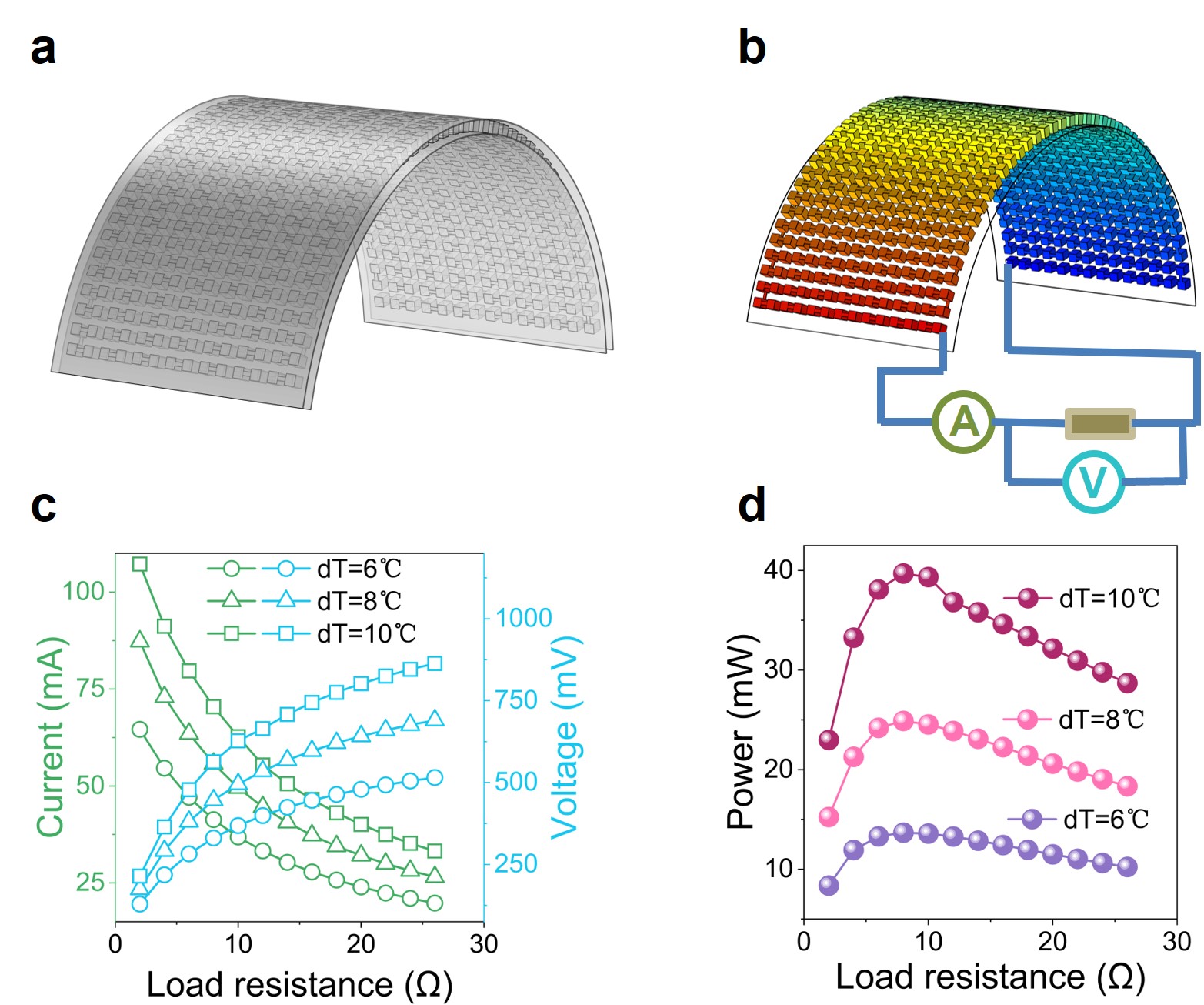


Figure S4. Thermoelectric calculation model of the FTEG. (a) Bending model. (b) Schematic diagram of the circuit node to connect the ammeter, the external load, the FTEG, and the voltmeter. (c) Generated voltage and current of the FTEG as a function of the external load resistance under different temperature gradients. (d) Generated power of the FTEG as a function of the external load resistance under different temperature gradients.


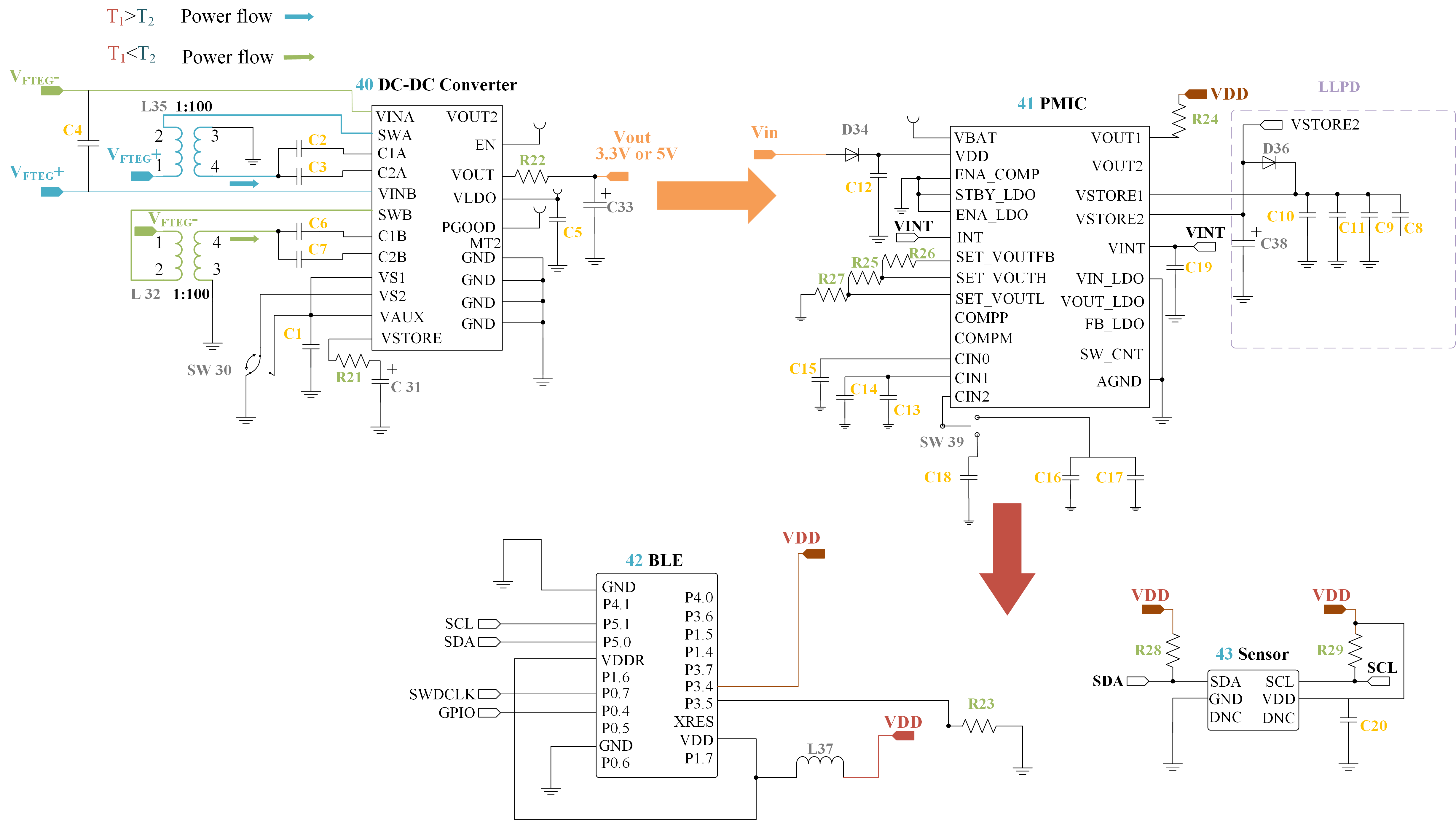


Figure S5. Schematic of the EM electronics.


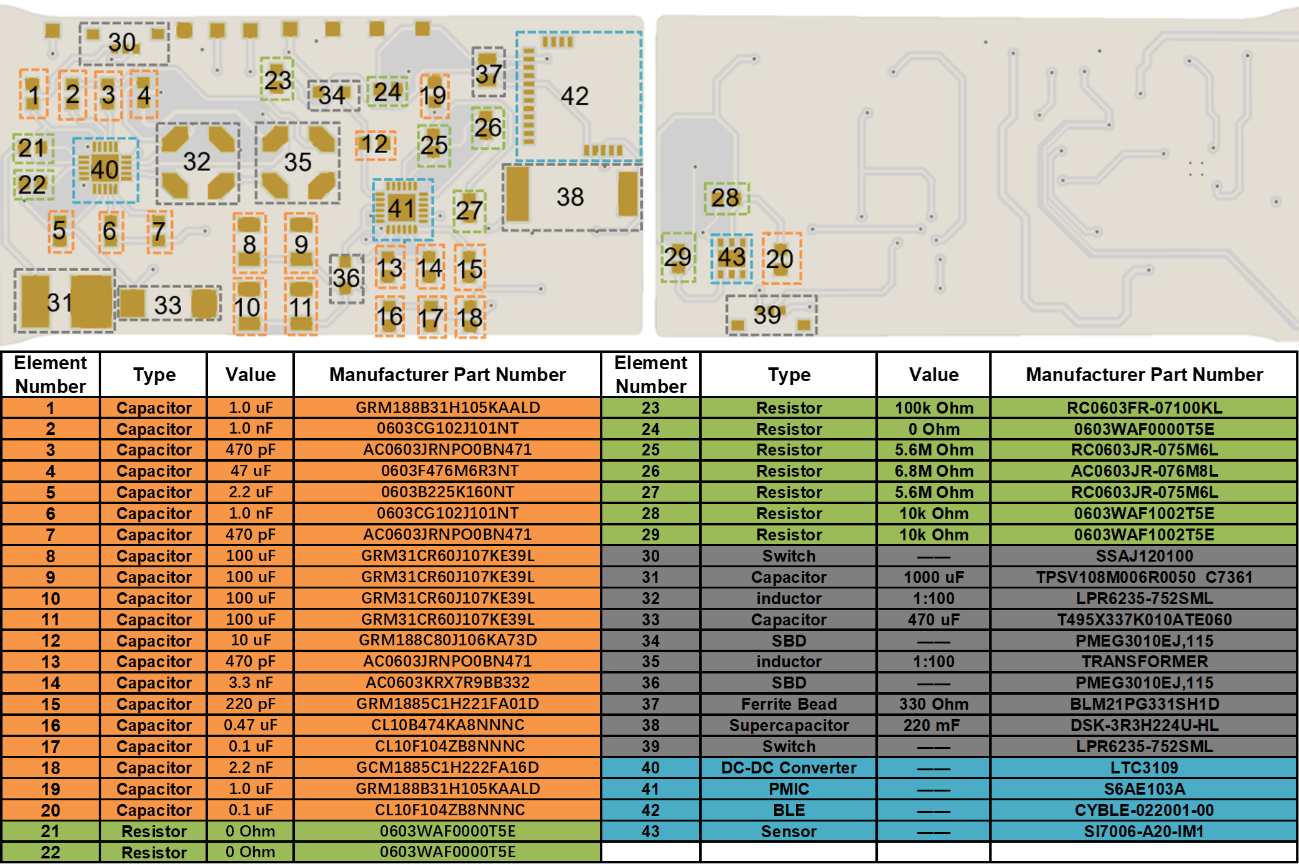


Figure S6. Layout of the EM electronics and bills of materials.


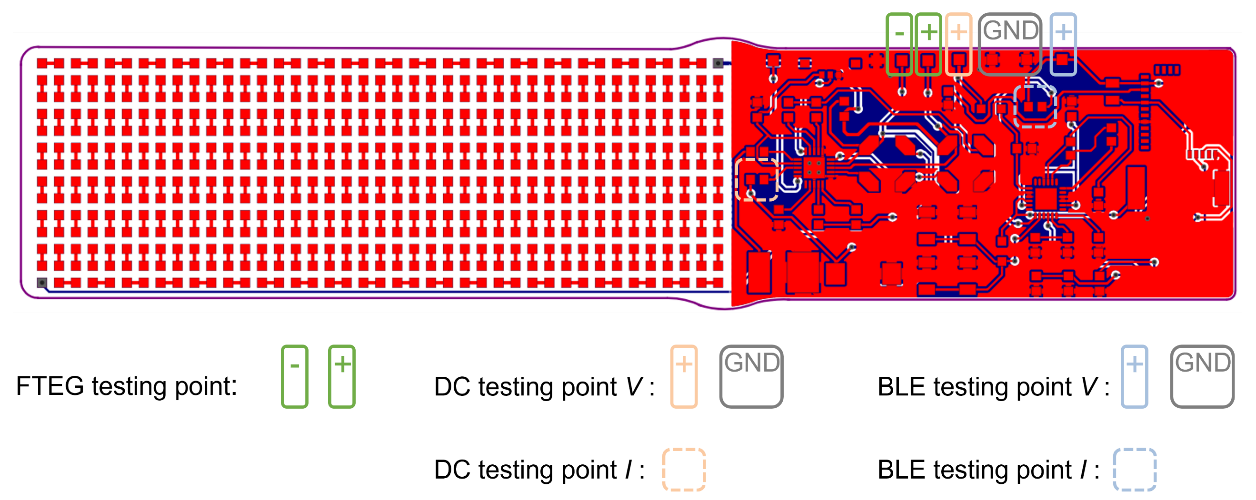


Figure S7. Characterization and testing of thermoelectric energy management electronics.


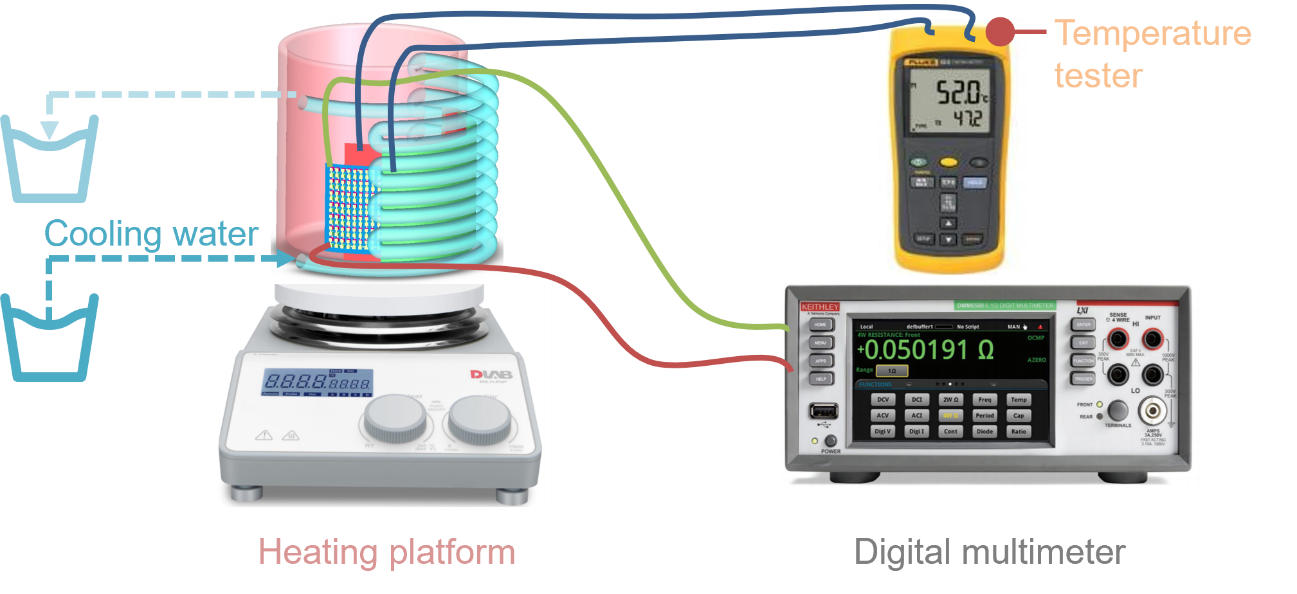


Figure S8. Illustration of the wearability testing setup for the FTEG.


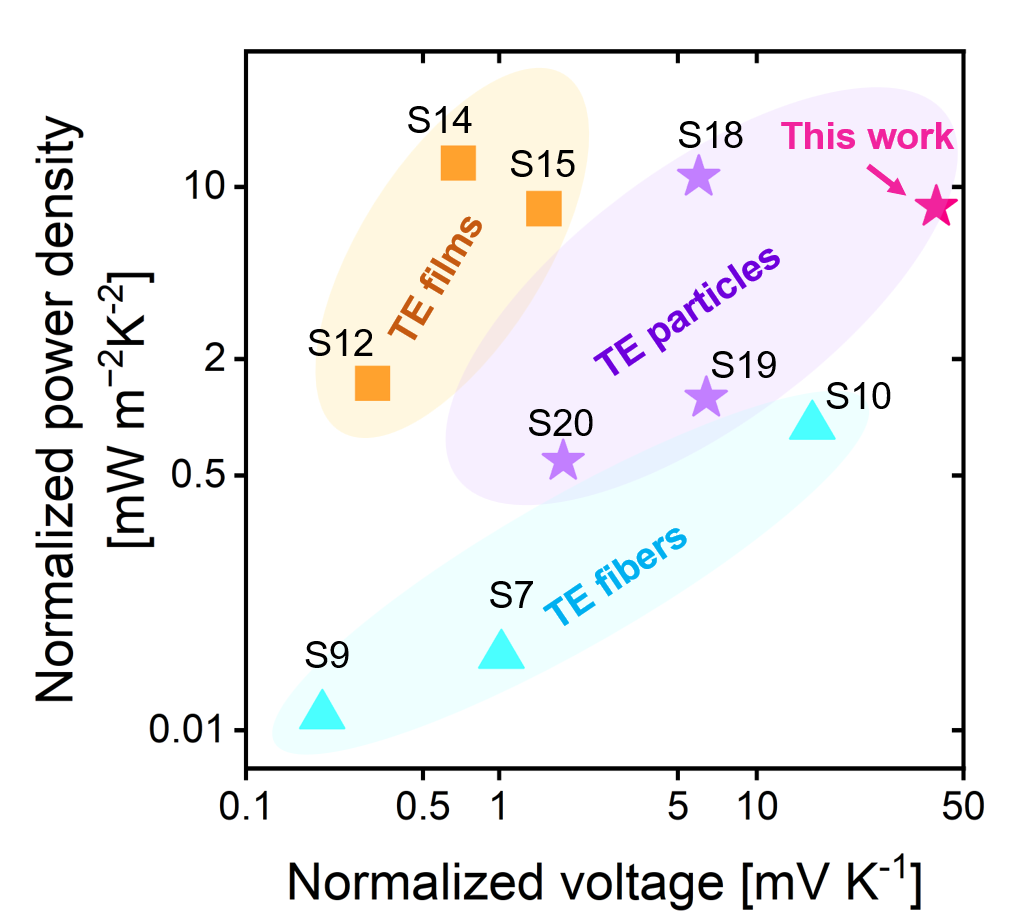


Figure S9. Performance comparison of different types of thermoelectric devices.

Table S1 Summary of the performance and application of TEGs

| **Ref.** | **Device** | **Number of thermocouples** | **Substrate** | **ΔT**  **(K)** | **Output voltage**  **(mV)** | **Power density** | **Applications** |
| --- | --- | --- | --- | --- | --- | --- | --- |
| S1 | AF-TEG  (3-level) | / | CNT | 13 | 350 | 8.5 W/m^3^ | LED |
| S2 | W-TEG+  Li-S battery | 96 | FPCB | 8.6 | 60 | 8.7 µW/cm^2^ | Charging the battery |
| S3 | S-TEG | 50 | Ecoflex elastomer | 19 | 123 | 170 uW/cm^2^ | Force sensor |
| S4 | S-TEG | 96 | / | 15 | 78.2 | 13.8 μW/cm^2^ | LED |
| S5 | C-TEGs | 220 | PDMS | 13 | 668 | / | LED |
| S6 | F-TEG | 50 | / | 9 | 50 | 10 μW/cm^2^ | LED |
| This work | FTEG+PM | 287 | PI | 4 | 167.5 | 13.6 μW/cm^2^ | BLE+Sensor |

Table S2 Classification of TEGs and their thermoelectric properties

| **Ref.** | **Materials** | **Generator types** | **ΔT [ K ]** | **Vmax [ mV ]** | **PDmax [ W m^−2^ ]** | **PDmax /ΔT^2^**  **[ mW m^−2^ K^-2^ ]** |
| --- | --- | --- | --- | --- | --- | --- |
| S7 | CNT/PEDOT: PSS fibers | TE fibers | 44 | ***45*** ^#^ | 0.070 | 0.036 |
| S8 | CNT/PEDOT: PSS nanofibers | TE fibers | 28 | 8.6 | — | — |
| S9 | CNT/PEDOT: PSS | TE fibers | 41 | 8.24 | 0.022 | 0.013 |
| S10 | (BTS)/PI/PDMS | TE fibers | 25 | ≈***400*** ^#^ | 0.58 | 0.928 |
| S11 | Ag_2_Se/PVP | TE films | 29.1 | ≈29 | 28.8 | 34.01 |
| S12 | PEDOT: PSS/SWCNTs | TE films | 20 | 6.34 | 0.609 | 1.52 |
| S13 | Ag_2_Se/PI | TE films | 30 | ≈17.5 | 29.55 | 32.83 |
| S14 | Ag/Ag_2_Se/PI | TE films | 27 | 18.5 | 8.74 | 11.99 |
| S15 | Cu_2_Se/Ag_2_Se/Ag_2_Se | TE films | 40 | ***60*** ^#^ | 13.4 | 8.38 |
| S16 | Bi_0.5_Sb_1.5_Te_3_\Bi_2_Te_2.7_Se_0.3_\elastic fabric | TE particles | 33.24 | ***111.49*** ^#^ | — | — |
| S17 | n-Bi_2_Te_3_/p-Sb_x_Te_y_/PI | TE particles | 29.9 | — | 143 | 160 |
| S18 | Bi_0.5_Sb_1.5_Te_3_/Bi_2_Se_0.5_Te_2.5_/PI | TE particles | 50 | — | 27 | 10.8 |
| S19 | p-Bi_2_Te_3_/n-Bi_2_Te_3_/PI | TE particles | 10.9 | ***70*** ^#^ | 0.1533 | 1.29 |
| S20 | p-Bi_2_Te_3_/n-Bi_2_Te_3_ | TE particles | 60 | ***107.8*** ^#^ | 2.36 | 0.6 |
| This work | Bi_2_Te_3_/PI/PDMS | TE particles | 4 | ***167*** ^#^ | 0.136 | 8.5 |

^#^ The output voltage of these devices (italic bold) surpasses the startup voltage (30 mV), making them suitable for utilizing EM electronics in this study.

Reference

[S1] Wu, B. et al. From carbon nanotubes to highly adaptive and flexible high-performance thermoelectric generators. Nano Energy 89, 106487 (2021).

[S2] Kim, J. et al. Self-charging wearables for continuous health monitoring. Nano Energy 79, 105419 (2021).

[S3] Yang, Y. et al. Stretchable Nanolayered Thermoelectric Energy Harvester on Complex and Dynamic Surfaces. Nano Letters 20, 4445-4453 (2020).

[S4] Fan, W. et al. High-Performance Stretchable Thermoelectric Generator for Self-Powered Wearable Electronics. Advanced Science 10, 2206397 (2023).

[S5] Lee, B. et al. High-performance compliant thermoelectric generators with magnetically self-assembled soft heat conductors for self-powered wearable electronics. Nat Commun 11, 5948 (2020).

[S6] Xu, Q. et al. High-performance, flexible thermoelectric generator based on bulk materials. Cell Reports Physical Science 3, 100780 (2022).

[S7] Sun, T. et al. Stretchable fabric generates electric power from woven thermoelectric fibers. Nature Communications 11, 572 (2020).

[S8] He, X. et al. Continuous manufacture of stretchable and integratable thermoelectric nanofiber yarn for human body energy harvesting and self-powered motion detection. Chemical Engineering Journal 450, 137937 (2022).

[S9] Wang, K., Hou, C., Zhang, Q., Li, Y. & Wang, H. Highly integrated fiber-shaped thermoelectric generators with radially heterogeneous interlayers. Nano Energy 95, 107055 (2022).

[S10] Zheng, Y. et al. Durable, stretchable and washable inorganic-based woven thermoelectric textiles for power generation and solid-state cooling. Energy & Environmental Science 15, 2374-2385 (2022).

[S11] Jiang, C. et al. Ultrahigh performance polyvinylpyrrolidone/Ag2Se composite thermoelectric film for flexible energy harvesting. Nano Energy 80, 105488 (2021).

[S12] Zhang, L. et al. Achieving high thermoelectric properties in PEDOT:PSS/SWCNTs composite films by a combination of dimethyl sulfoxide doping and NaBH4 dedoping. Carbon 196, 718-726 (2022).

[S13] Lei, Y. et al. Microstructurally Tailored Thin β-Ag2Se Films toward Commercial Flexible Thermoelectrics. Advanced Materials 34, 2104786 (2022).

[S14] Gao, Q. et al. High Power Factor Ag/Ag2Se Composite Films for Flexible Thermoelectric Generators. ACS Applied Materials & Interfaces 13, 14327-14333 (2021).

[S15] Xie, J. et al. Flexible pCu2Se-nAg2Se thermoelectric devices via in situ conversion from printed Cu patterns. Chemical Engineering Journal 435, 135172 (2022).

[S16] Hou, Y. et al. Whole Fabric-Assisted Thermoelectric Devices for Wearable Electronics (Adv. Sci. 1/2022). Advanced Science 9, 2270002 (2022).

[S17] Zhang, J. et al. Flexible micro thermoelectric generators with high power density and light weight. Nano Energy 105, 108023 (2023).

[S18] Shi, Y. et al. Stretchable thermoelectric generator for wearable power source and temperature detection applications. Energy Conversion and Management 253, 115167 (2022).

[S19] Jeong, M.H., Kim, K.-C., Kim, J.-S. & Choi, K.J. Operation of Wearable Thermoelectric Generators Using Dual Sources of Heat and Light. Advanced Science 9, 2104915 (2022).

[S20] Zadan, M. et al. Liquid Crystal Elastomer with Integrated Soft Thermoelectrics for Shape Memory Actuation and Energy Harvesting. Advanced Materials 34, 2200857 (2022).
